# Identification of epigenetic silencing of the *SFRP2* gene in colorectal cancer as a clinical biomarker and molecular significance

**DOI:** 10.1186/s12967-024-05329-x

**Published:** 2024-05-27

**Authors:** Hatim Boughanem, Jesús pilo, Libia Alejandra García-Flores, Isabel Arranz, María Ramos-Fernandez, María Ortega-Castan, Ana B. Crujeiras, Juan Sandoval, Manuel Macias-Gonzalez

**Affiliations:** 1 Department of Endocrinology and Nutrition, Virgen de la Victoria University Hospital, Malaga, Spain; 2 Institute of Biomedical Research in Malaga (IBIMA)-Bionand Platform, University of Malaga, 29010 Malaga, Spain; 3 Spanish Biomedical Research Center in Physiopathology of Obesity and Nutrition (CIBERObn), Instituto de Salud Carlos III, Madrid, Spain; 4 Unidad de Gestión Clinica Medicina Interna, Lipids and Atherosclerosis Unit, Maimonides Institute for Biomedical Research in Córdoba, Reina Sofia University Hospital, University of Córdoba, 14004 Córdoba, Spain; 5 Division of Anatomical Pathology, Hospital Universitario Virgen de la Victoria, 29010 Malaga, Spain; 6 Department of Human Physiology, Human Histology, Anatomical Pathology and Physical Education, University of Malaga, 29010 Malaga, Spain; 7 Unidad de Gestion Clinica Cirugía General y del Aparato Digestivo, Virgen de la Victoria University, 29010 Malaga, Spain; 8 Epigenomics in Endocrinology and Nutrition Group, Epigenomics Unit, Instituto de Investigación Sanitaria de Santiago de Compostela (IDIS), Complejo Hospitalario Universitario de Santiago de Compostela (CHUS/SERGAS), 15706 Santiago de Compostela, Spain; 9 Epigenomics Core Facility and Biomarkers and Precision Medicine Unit, Health Research Institute La Fe, 46026 Valencia, Spain

**Keywords:** *SRFP2*, DNA methylation, Colorectal cancer biomarkers, Promoter methylation, Wnt signaling pathway

## Abstract

**Background:**

Several studies have suggested secreted frizzled-related protein 2 (*SFRP2*) gene as a potential clinical biomarker in colorectal cancer (CRC). However, its diagnostic role remains unclear. In this study, we aimed to investigate the significance of *SFRP2* methylation levels in a large cohort of biological specimens (including blood, adipose and colonic tissues) from patients with CRC, thereby potentially identifying new biomarker utility.

**Methods:**

We examined the expression (by qPCR) and methylation status (by 450 K DNA array and DNA pyrosequencing) of the *SFRP2* gene in healthy participants (N = 110, aged as 53.7 (14.2), 48/62 males/females) and patients with CRC (N = 85, aged 67.7 (10.5), 61/24 males/females), across different biological tissues, and assessing its potential as a biomarker for CRC. Additionally, we investigated the effect of recombinant human SFRP2 (rhSFRP2) as a therapeutic target, on cell proliferation, migration, and the expression of key genes related to carcinogenesis and the Wnt pathway.

**Results:**

Our findings revealed that *SFRP2* promoter methylation in whole blood could predict cancer stage (I + II vs. III + IV) (AUC = 0.653), lymph node invasion (AUC = 0.692), and CRC recurrence (AUC = 0.699) in patients with CRC (all with *p* < 0.05). Furthermore, we observed a global hypomethylation of *SFRP2* in tumors compared to the adjacent area (*p* < 0.001). This observation was validated in the TCGA-COAD and TCGA-READ cohorts, demonstrating overall hypermethylation (both with *p* < 0.001) and low expression (*p* < 0.001), as shown in publicly available scRNA-Seq data. Notably, neoadjuvant-treated CRC patients exhibited lower *SFRP2* methylation levels compared to untreated patients (*p* < 0.05) and low promoter *SFRP2* methylation in untreated patients was associated with poor overall survival (*p* < 0.05), when compared to high methylation. Finally, treatment with 5 µg of rhSFRP2 treatment in CRC cells (HCT116 cells) inhibited cell proliferation (*p* < 0.001) and migration (*p* < 0.05), and downregulated the expression of *AXIN2* (*p* < 0.01), a gene involved in Wnt signaling pathway.

**Conclusions:**

These findings establish promoter methylation of the *SFRP2* gene as a prognostic candidate in CRC when assessed in blood, and as a therapeutic prognostic candidate in tumors, potentially valuable in clinical practice. SFRP2 also emerges as a therapeutic option, providing new clinical and therapeutical avenues.

**Supplementary Information:**

The online version contains supplementary material available at 10.1186/s12967-024-05329-x.

## Introduction

Colorectal cancer (CRC) is the third most common cancer and the fourth leading cause of cancer associated mortality. By 2040, it is estimated that the incidence of CRC will increase up to 63% increase, reflecting a 73% rise in mortality rates [1]. The overall 5-year survival rate for CRC stands at 63%. However, diagnosis at early and localized stage rises the survival rate up to 91%. Nevertheless, advanced cases decrease drastically the overall survival, up to 12–14% [2, 3], clearly positioning CRC as a major public health problem.

Generally, CRC originates from genetic and epigenetic alterations in colonic epithelial cells [4, 5], which play a pivotal role in both carcinogenesis and cancer progression. Epigenetic alterations in cancer are considered the hallmark of human tumors. Specifically, DNA methylation is the most significant epigenetic alterations in CRC, mainly affecting promoter regions of tumor suppressor genes, leading to their inactivation and contributing to uncontrolled cell proliferation [6]. In particular, epigenetic alterations in the secreted frizzled-related protein 2 (*SFRP2*) gene have been proposed as a potential biomarker in CRC.

SFRP2 is an extracellular Wnt antagonist that binds to Wnt ligands, and inhibit the Wnt signaling. This inhibition prevents β-catenin from translocating from the cytoplasm to the nucleus, affecting cell proliferation, survival, differentiation, and migration [7]. The latest meta-analysis conducted by Yu et al. (2019), which included 83 studies with a total of 21,612 samples, identified a significant association between *SFRP2* promoter hypermethylation (in different biological tissues) and an increased risk of CRC, with an overall pooled odds ratio (OR) of 8.41 (95% CI 5.91–11.97) [8]. Another meta-analysis, conducted by Shariatpanahi et al., found that promoter methylation of *SFRP2* in stool samples was associated with an increased risk of CRC (OR = 35.3 [18.7–66.8], AUC = 0.94) [9], indicating its potential use as a diagnostic biomarker. However, the sensitivity of *SFRP2* methylation as a biomarker for CRC varies widely. Moreover, the level of *SFRP2* methylation differs among different tissues. While *SFRP2* methylation in stool samples can be useful for cancer diagnosis, *SFRP2* methylation in blood samples may serve as a prognostic factor [10]. This variability can be attributed to various factors that may disrupt *SFRP2* methylation status. In particular, it has been reported that *SFRP2* methylation is significantly higher on the right side of CRC (57.33%) compared to the left side (36.60%) [11]. Therefore, assessing *SFRP2* methylation in other tissues may serve as a stable prognostic factor.

Nevertheless, there are limited studies investigating the potential of *SFRP2* methylation as a predictor for other cancer outcomes, such as response to therapy or recurrence in other biological and less invasive tissues. This highlights the need for further research to identify its predictive value. Additionally, there are few studies concerning the use of recombinant human SFRP2 (rhSFRP2) in cancer research, emphasizing the urgency of exploring its potential as a therapeutic target. Therefore, the aim of this study is to test whether there is a significant association between *SFRP2* DNA methylation and other cancer outcomes, such as survival rates, response to therapy, location, or recurrence in blood and tumor samples. Furthermore, we aimed to evaluate the effect of recombinant human SFRP2 in colorectal cancer cell model (HCT116 cells) to understand its contribution to colorectal carcinogenesis, and to recommend new strategies for its use as a cancer biomarker.

## Materials and methods

### Participants and study design

This study recruited participants from the “Virgen de la Victoria” University Hospital between 2012 and 2014. A total of 110 healthy participants (without CRC) were included, who underwent hiatus hernia surgery or cholecystectomy, which was the method used to obtain adipose tissue. Additionally, 85 patients diagnosed with CRC were included, diagnosed by a pathology specialist using a biopsy and colonoscopy. Biopsy samples were classified according to histological features by pathologists, according to the “World Health Organization Classification of Tumors of the Digestive System” (2016) [12]. Additional medical records and pathological examinations were reviewed to confirm the diagnosis. All patients with CRC underwent surgery with curative intent, specifically, a hemicolectomy and lower anterior resection with an ileostomy, followed by total meso-colorectal excision. For rectal cases, total mesorectal excision preceded. Patients with CRC were treated with radiation therapy and/or fluoropyrimidine-based chemotherapy. All patients had at least a 5-year follow-up, including clinical visits, checkups, and biochemical variables every three months for the first two years, then every six months starting in the third year. A colonoscopy was performed annually. Exclusion criteria were patients with inflammatory and cardiovascular diseases, hereditary non-polyposis colorectal cancer or familial adenomatous polyposis, type 2 diabetes, insulin resistance, or renal and infectious diseases. Participants who had undergone treatment that altered lipid or glucose metabolism or who consumed > 20 g of ethanol per day were excluded. Written informed consent was obtained from all patients and subjects and reviewed and approved by the Ethics Committees of the “Virgen de la Victoria” University Hospital (Málaga, Spain) (Registration Number: 0311/PI7). The study was conducted in accordance with the guidelines laid down in the Declaration of Helsinki.

### Samples included in the study

Blood samples were collected from all participants of the same patients, and serum samples were obtained by centrifugation of the blood samples at 4000 rpm for 15 min at 4 °C. Epiploic adipose tissue samples (surrounding and attached to the colon) were obtained during surgery and immediately frozen in liquid nitrogen for transportation to the laboratory. The adipose tissue samples were then washed with PBS, cut into 100 mg pieces, and stored at −80 °C until processing. Biopsy samples were used to obtain tumor samples containing both the tumor area and the adjacent normal tumor-free area (NAT). These samples were fixed using formalin and embedded in paraffin (formalin-fixed paraffin-embedded or FFPE). Pathologists determined the limits between the areas and calculated the percentage of tumor cells within the tumor area, which was greater than 80% of the tumor cell percentage.

### Biochemical variables determination

Fasting glucose, total cholesterol, triglycerides, and high-density lipoprotein cholesterol (HDL-c) were measured using the Dimension Autoanalyzer (Dade Behring Inc., USA). Low-density lipoprotein cholesterol (LDL-c) was calculated using the Friedewald equation [13]. Insulin levels were determined by radioimmunoassay methods using BioSource International Inc. (Camarillo, CA, USA). The homeostasis model assessment of insulin resistance (HOMA-IR) was calculated using the following equation: HOMA-IR = fasting insulin (μIU/mL) x fasting glucose (mmol/L)/22.5 [14].

### DNA and RNA extraction

Genomic DNA was extracted from 200 µl of blood samples and HCT116 cells (1 × 10^4^–1 × 10^5^ cells) using the Qiamp DNA mini-Kit (Qiagen GmbH, Hilden, Germany), following the manufacturer's instructions. For adipose tissue, 30 mg of tissue were used, and genomic DNA was extracted using the Qiamp DNA Tissue Kit (Qiagen GmbH, Hilden, Germany) according to the manufacturer's instructions. Genomic DNA from the tumor and NAT area was isolated from 5 to 10 sections of 14 μm of FFPE samples, using the Qiamp DNA FFPE Tissue Kit according to the manufacturer's instructions (Qiagen GmbH, Hilden, Germany). DNA integrity was determined using the NanoDrop ND-1000 (Thermo Fisher Scientific, Indianapolis, USA) at 260/280 and 260/230 ratios and confirmed by electrophoresis in a 1.5% agarose gel in TAE buffer. The Pico Green dsDNA Quantitation Reagent (Invitrogen, Carlsbad, CA) was used to further analyze DNA integrity and quantify DNA concentrations.

For RNA extraction, total RNA was isolated from HCT116 cells (at a density of 1 × 10^4^–1 × 10^5^ cells) using the Total RNA Purification Kit (Norgen Biotek Corp., ON, Canada) according to the manufacturer’s instructions. Total RNA from blood and adipose tissue samples was isolated using the Qiamp RNA Easy Kit (Qiagen GmbH, Hilden, Germany) and the RNeasy Lipid Tissue Mini Kit (Qiagen GmbH, Hilden, Germany), respectively, according to the manufacturer's instructions. Total RNA from FFPE tumor samples was isolated from 5 to 10 sections of 14 μm using the RNeasy FFPE Kit, following the manufacturer's instructions (Qiagen GmbH, Hilden, Germany). RNA quantity and integrity was determined using the NanoDrop ND-1000 (Thermo Fisher Scientific, Indianapolis, USA) at 260/280 and 260/230 ratios. To generate first-strand cDNA synthesis, 1 μg of total extracted RNA and random primers were used with the PrimeScript^™^ RT-PCR Kit as indicated by the manufacturer.

### Bisulfite reaction and genome-wide DNA methylation analysis

For the genome-wide methylation analysis study, high-quality genomic DNA samples (500 ng) from the tumor area (N = 27) and the NAT area (N = 15) were treated with bisulfite using the EZ-96 DNA Methylation Kit (Zymo Research, Irvine, CA), following the manufacturer's instructions. Subsequently, DNA methylation was analyzed by microarray assays using Infinium Human Methylation 450 K bead chip technology (Illumina, San Diego, CA). Whole-genome amplification and hybridization were then performed using BeadChip, followed by single-base extension and analysis using the HiScan SQ module (Illumina) to assess cytosine methylation states. DNA quality checks, bisulfite modification, hybridization, data normalization, statistical filtering, and value calculation were performed as previously described [15]. For the pyrosequencing reaction, purified DNA (2 μg) from different tissues was used for the bisulfite reaction, using the EpiTect Fast Bisulfite Kit (Qiagen GmbH, Hilden, Germany). The primer sequences for the promoter of the *SFRP2* gene were as follows: forward primer: AGAAGTTTTGGGTTTAGTTTATGAT, and reverse primer: CTCACATCTACCCAATATAAAAACTCACCA. A PCR reaction was performed using 0.2 nmol/L of primers. DNA pyrosequencing was carried out using the PyroMark Q96 ID pyrosequencing System (Qiagen GmbH, Hilden, Germany) and the sequencing primer: TGTTGAAYGGTGGTTGG. The methylation average was presented as the percentage of methylated cytosine over the sum of methylated and unmethylated cytosines. Interassay precision (%CV) was 2.5%, and intraassay precision (%CV) was 1.0%. Non-CpG cytosine residues were used as built-in controls to verify bisulfite conversion. We also used unmethylated and methylated DNA as controls in our assay (New England Biolabs, UK).

### Gene expression analysis

For the quantification of SFRP2 (Hs.PT.58.20705989), AXIN2 (Hs.PT.58.39305692), CTNNB1 (beta-catenin) (Hs.PT.58.40551289), TIAM1 (Tumor Growth Factor beta 1) (Hs.PT.58.24232612), VEGF (Vascular Endothelial Growth Factor) (Hs.PT.58.21234833) (all related with the Wnt pathway) and *PPIA* (Peptidylprolyl isomerase A) (Hs.PT.58v.38887593.g) genes, we used commercially available TaqMan primer/probe mix (Integrated DNA Technologies Inc., Madrid, Spain). Gene expression was quantified using Premix Ex Taq^™^ (Probe qPCR) (Takara Bio USA, Inc., Mountain View, CA), following the manufacturer's instructions on the QuantStudio 6 Pro (Applied Biosystems, Darmstadt, Germany). Gene expression was normalized by the 2^−ΔCt^ method [16], expressed as the target gene/*PPIA* ratio.

### Cell culture

HCT116 (obtained from ATCC with the reference: CCL-247^™^) human colorectal carcinoma cells were cultured in Dulbecco’s Modified Eagle’s Medium—low glucose (Biowest, France), plus 10% fetal bovine serum (FBS) (Gibco, CA, USA), 1% of l-glutamine and 1% of streptomycin/penicillin at 37 °C and 5% CO_2_. Cells were periodically checked for mycoplasma using DAPI analysis [17] and PCR using universal primers [18]. Cells are maintained at passages lower than 20. All cell experiments were conducted in triplicates and three independent replicates.

### DNA demethylation in vitro

For the 5-Aza-2-deoxycytidine (AZA) (A3656, Sigma Aldrich, Madrid, Spain) treatment as an inhibitor of DNA methyltransferases (DNMTs), cells were treated with 1 µM, 5 µM, and 10 µM of AZA for 72 h (increased concentration to test if the effect is dose-dependent). The media was renewed every 48 h, and RNA extraction was performed after 72 h of treatment.

### Cell proliferation assay

Cells were seeded in 96-well plates at a concentration of 5,000 cells/well. After 24 h, the cells were treated with 0.1, 0.5, 1, 2.5, and 5 µg/mL of rhSFRP2 (recombinant human SFRP2) for 48 h (to find the concentration that cause decrease in cell proliferation). After the treatment, the media were removed, and the MTT reagent was added using an MTT Assay Kit (Cell Proliferation, Cat. No. ab211091, Abcam, UK) and incubated for 3 h at 37 ºC. Then, the crystals were dissolved in the solvent reagent, and the absorbance at 590 nm was measured.

### Migration assay

A 96-well tissue culture plate was seeded with cells at a density of 50,000 cells/well. After 24 h, a circular wound was created by scratching the monolayer as described in [19]. A p10 pipette tip attached to a vacuum pump was gently pressed perpendicularly onto the cell monolayer, without lateral movement, to detach cells from the substratum and create a circular wound. Cells were then treated with 5 µg/mL of rhSFRP2, and images were captured at 0, 24, and 48 h. Migration was quantified by calculating the gap distance and expressing it as a percentage of control (i.e., 100% wound healing). The gap distance was measured using ImageJ software (http://rsb.info.nih.gov/ij/download.html).

### Bioinformatic analysis

For DNA methylation analysis, we used the minfi package and the ChAMP pipeline from Bioconductor with default settings [20, 21]. Probes with p-values above 0.01 were excluded, and normalization and preprocessing were conducted using the minfi package. Probes were stratified by region and bad samples (low intensity) were eliminated using the median of methylated and unmethylated signals for each sample. Probes with single nucleotide polymorphisms at CpG or single-base extension sites and those on sex chromosomes were removed, and M values were obtained for further investigation.

For TCGA data analysis, we used the “*TCGAMethylation450k*” and “*TCGAbiolinks*” packages to extract data from The Cancer Genome Atlas (TCGA) database (Cancer Genome Atlas Research) using mainly *idat* files and information of participants. We obtained methylation data from June 2023, for TCGA-COAD (colon adenocarcinoma) and TCGA-READ (rectal adenocarcinoma) from The Cancer Genome Atlas (TCGA) database (Cancer Genome Atlas Research) (with the primary objective to validate and assess the consistency of our results, as well as increase confidence in the robustness and reproducibility of our findings) [22]. We included IDAT files from 308 cancer cases and 41 NAT from TCGA-COAD, and 94 cancer cases and 10 NAT from TCGA-READ, which were downloaded and processed using the “*TCGAbiolinks”* R packages [23]. The data were filtered, normalized, and preprocessed following similar protocols as described above (probes with p-values exceeding 0.01 were omitted). Survival analyses were extracted from the OncoDB web tool [24]. We used normalized RNA-sequencing (RNA-seq) expression data from TCGA-COAD and TCGA-READ, obtained using the TCGAbiolinks R package, to identify differentially expressed genes. For the single-cell analysis, we extracted the results from single-cell analysis using 52,609 cells, in June 2023, as published by Lee HO et al. [25], using the EMBL-EBI platform: https://www.ebi.ac.uk/

### Statistical analysis

The results are presented as mean ± standard deviation (SD) for continuous variables and as numbers (percentages) for categorical variables. Depending on the normality of the variables, either a Student’s t-test or a Wilcoxon test was used. Pearson correlation coefficients were calculated to evaluate the associations between methylation and anthropometric and biochemical parameters. Overall survival analyses were conducted using Kaplan–Meier curves, and multivariate proportional regression was used to calculate odds ratios (ORs) [95% confidence intervals (CIs)] and area under curve (AUC) for *SFRP2* methylation adjusted by age, sex, BMI, HDL, triglycerides and cancer location. Logistic and linear regression analyses were conducted using SFRP2 promoter methylation in tumor, adjusted for age, sex and BMI. All analyses were performed in R version 3.5.1, and statistical significance was set at *p* < 0.05 [26]. Graphical representations were also created using R.

## Results

### General clinical and characteristic data of the participants

The baseline biochemical and anthropometric data of healthy participants and patients with CRC are summarized in Table 1. As observed, patients with CRC were older than healthy participants (*p* < 0.001). Furthermore, glucose and triglyceride levels were higher in patients with CRC compared to healthy participants (p = 0.004 and p = 0.032, respectively). In contrast, HOMA-IR, total cholesterol, LDL-c and HDL-c levels were lower in patients with CRC than in healthy participants (all with *p* < 0.001). Importantly, there was no significant difference in BMI when comparing both groups.

**Table 1:** Baseline table of the participants included in the study

Variables	All	Healthy participants	Patients with CRC	*p*
	*N* = 195	*N* = 110	*N* = 85	
Age (years)	59.9 (14.5)	53.7 (14.2)	67.7 (10.5)	< 0.001*
Sex (Males/Females)	109/86	48/62	61/24	< 0.001*
BMI (Kg/m^2^)	27.7 (4.17)	28.2 (4.28)	27.2 (4.00)	0.121
Glucose (mg/dL)	104 (17.1)	101 (16.3)	108 (17.4)	0.004*
HOMAIR	2.05 (1.29)	2.38 (1.27)	1.60 (1.17)	< 0.001*
Total cholesterol (mg/dL)	196 (47.2)	214 (44.1)	173 (40.8)	< 0.001*
Triglycerides (mg/dL)	135 (49.2)	129 (47.9)	144 (49.9)	0.032*
HDL (mg/dL)	46.7 (13.2)	51.6 (11.9)	40.7 (12.2)	< 0.001*
LDL (mg/dL)	120 (35.3)	133 (31.3)	103 (33.2)	< 0.001*

Data are expressed as mean ± standard deviations or percentage.

*BMI* Body mass index, *CRC* colorectal cancer, *HOMAIR* homeostasis model of insulin resistance, *HDL* High-density lipoprotein, *LDL* Low-density lipoprotein

Asterisk indicates significant difference between groups, according to Welch’s two sample test (*p < 0.05). Chi squared test was used for variables expressed as percentage (*p < 0.05)

### DNA methylation of the SFRP2 promoter in whole blood as a biomarker in colorectal *cancer*

**Fig. 1:**
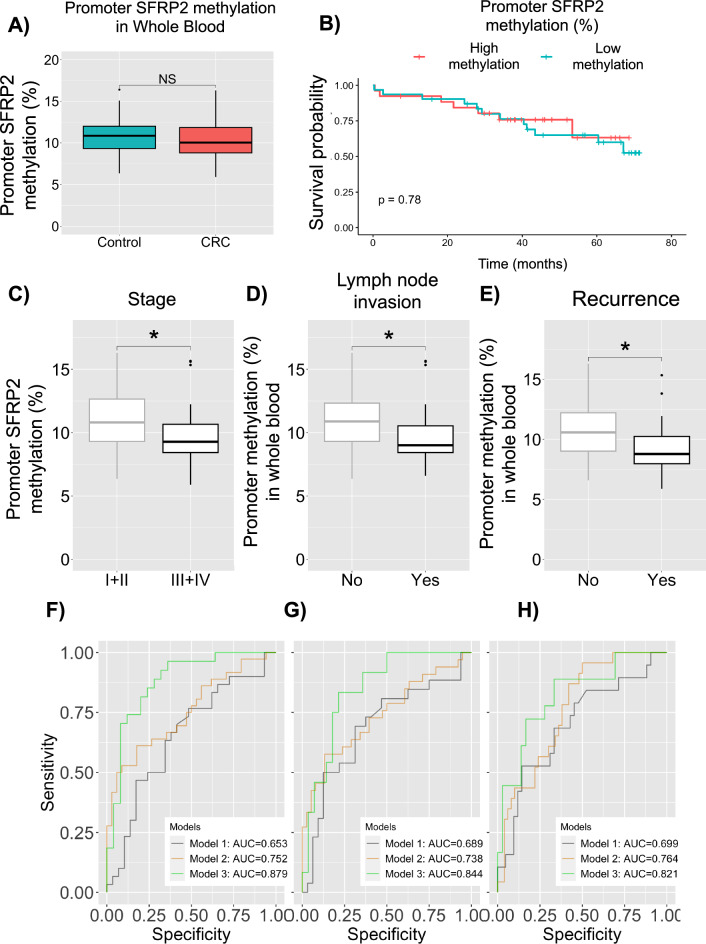
Promoter *SFRP2* methylation in whole blood as a biomarker for colorectal cancer. **A** We measured the promoter methylation of *SFRP2* gene from healthy participants (N = 31) and patients with CRC (N = 62) (p = 0.220). **B** Kaplan–Meier curve comparing the median of the *SFRP2* promoter methylation as low and high methylation. The significance of differences is evaluated with the Log-rank test. We measured the promoter methylation of *SFRP2* gene from patients with CRC in **C** early stage (I + II) (N = 42) and late stage (III + IV) (N = 39), (p = 0.044) **D** absence (N = 44) and presence (N = 35) of lymph node invasion (p = 0.014) and **E** absence (N = 58) and presence (N = 24) of recurrence (p = 0.013). Asterisks indicate significant differences between the groups according to the Mann Whitney test (*p < 0.05, **p < 0.01, ***p < 0.001). **F**, **G** and **H** indicate ROC curve of stage (**F**), lymph node invasion (**G**) and Recurrence (**H**) in three models. Model 1 indicate *SFRP2* methylation. Model 2 indicates clinical variables, such as age, sex, BMI, HDL, triglycerides, and tumor location. Model 3 includes Model 1 + Model 2. *AUC* Area under curve, *CRC* colorectal cancer, *ROC* receiver operating characteristic curve, *Sens* sensitivity, *Spec* specificity

In this study, we measured the expression and methylation levels of the *SFRP2* gene in whole blood and adipose tissue. As depicted in Fig. 1A, there were no significant differences in *SFRP2* promoter methylation levels analyzed by pyrosequencing between healthy participants and patients with CRC in whole blood. Additionally, we did not observe any significant differences in the prognostic value of promoter *SFRP2* methylation levels in whole blood between high and low methylation levels (Fig. 1B). Similar results were observed in *SFRP2* gene expression levels between healthy participants and patients with CRC in whole blood (Supplementary Figs. 1A and B).

When we examined other cancer outcomes, we found that the promoter *SFRP2* methylation levels were significantly lower in patients with CRC in late stage (III + IV) than in those in the early stage (I + II) (*p* = 0.044) (Fig. 1C), and also when compared with healthy participants (*p* = 0.035) (Supplementary Fig. 1C), with an AUC of 0.879 (95% CI 0.700–0.942) (Fig. 1F, Supplementary Table 1) when adjusted by clinical variables. Patients with CRC who had lymph node invasion also had lower *SFRP2* methylation levels than those without lymph node invasion (*p* = 0.014) (Fig. 1D), with an AUC of 0.844 (95% CI 0.736–0.951) (Fig. 1G, Supplementary Table 1). Finally, patients with CRC who had recurrence had lower *SFRP2* methylation levels than those without recurrence (*p* = 0.013) (Fig. 1E), with an AUC of 0.821 (95% CI 0.700–0.942) (Fig. 1H, Supplementary Table 1). We also measured the expression and methylation levels of the *SFRP2* gene in adipose tissue. As shown in Supplementary Fig. 1D, there were no significant differences in *SFRP2* promoter methylation levels between healthy participants and patients with CRC. However, patients with CRC had lower *SFRP2* expression levels than healthy participants (*p* = 0.002) (Supplementary Fig. 1F). Furthermore, we did not observe any significant differences between high and low *SFRP2* methylation or expression levels in adipose tissue (Supplementary Fig. 1E and Supplementary Fig. 1G).

### DNA Methylation of the SFRP2 gene in tumoral tissue from the same cohort as a biomarker in colorectal *cancer*

**Fig. 2:**
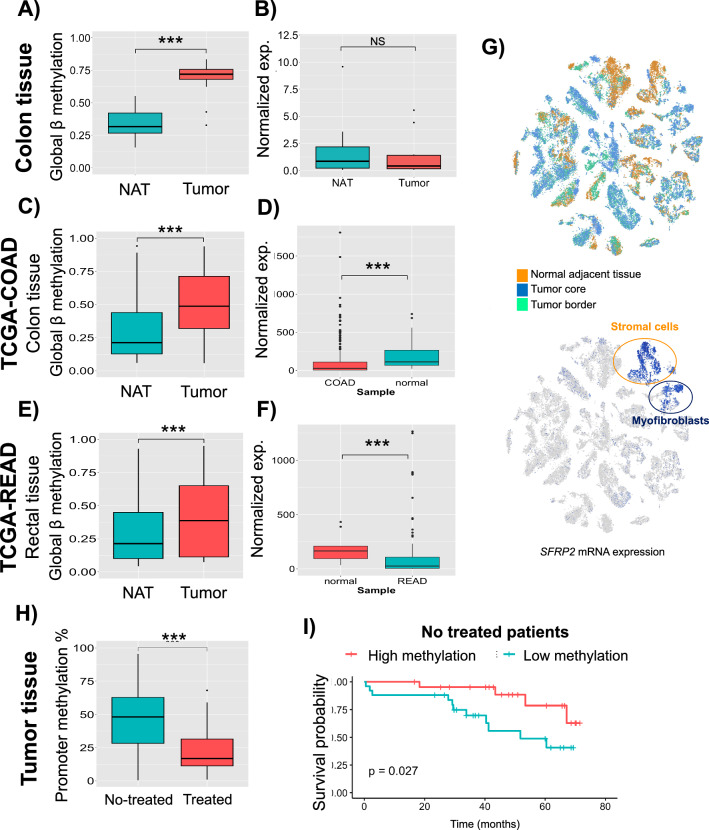
Diagnostic and prognostic value of SFRP2 methylation in tumor tissue from colorectal cancer. **A** Global β methylation was done with significant CpG sites between tumor and NAT. The CpG sites included in the global SFRP2 methylation in colon tissue in our participants were: cg14289246, cg05961809, cg23292160, cg25775322, cg23207990, cg22178613, cg10318528, cg11354906, cg00082664, cg03202804, cg05874561, cg04965141, cg25645268, cg14330641, cg23121156, cg06549216, cg10942078, cg20881942, cg23714408, cg05164933, cg14063488. **B** *SFRP2* expression in colon samples from NAT (N = 11) and tumor area(N = 16). **C** Global β methylation was done with significant CpG sites between tumor and NAT in the TCGA-COAD. **D** *SFRP2* expression in colon samples from NAT (N = 51) and tumor area (N = 308) in the TCGA-COAD cohort. **E** Global β methylation was done with significant CpG sites between tumor and NAT in the TCGA-READ. **F** *SFRP2* expression in colon samples from NAT (N = 10) and tumor area(N = 94) in the TCGA-READ cohort. **G** Single cell RNA-seq analysis of SFRP2 gene in NAT, tumor core and tumor border. SFRP2 was mostly expressed in NAT and a small fraction of tumor core, in stromal cells and myofibroblasts. **H** We measured the promoter methylation of *SFRP2* gene from patients with CRC no treated (N = 57) and treated (N = 24) with neoadjuvant therapy. **I** Kaplan–Meier curve comparing the median of the SFRP2 promoter methylation as low and high methylation in non-treated patients with CRC. The significance of differences is evaluated with the Log-rank test. Asterisks indicate significant differences between the groups according to the Mann Whitney test (*p < 0.05, **p < 0.01, ***p < 0.001). *COAD* colon adenocarcinoma, *NAT* normal adjacent area, *READ* rectum adenocarcinoma, *TCGA* The cancer genome atlas

We next evaluated the expression and methylation of the *SFPP2* gene in colonic tissue taking advantage of Infinium DNA methylation 450 K data (adjusted and age, sex and BMI). As shown in Fig. 2A, global β methylation of the *SFRP2* gene was higher in the tumor area, when compared to the NAT area (*p* < 0.001). However, we did not find a significant difference between both areas regarding *SFRP2* expression (*p* = 0.171) (Fig. 2B). After that, we next validated these findings in the TCGA-COAD and TCGA-READ. As a result, we discovered that global β methylation of the *SFRP2* gene was higher in the tumor area than in the NAT area (*p* < 0.001) in both TCGA-COAD and -READ (Fig. 2C, F). Furthermore, when we focused on the prognostic value of promoter *SFRP2* methylation in tumors and in the TCGA-COAD and TCGA-READ, we found no significant difference between high and low *SFRP2* methylation in global β methylation, promoter methylation, and in the TCGA-COAD and TCGA-READ (Supplementary Fig. 2A, Supplementary Fig. 2B, Supplementary Fig. 2C, Supplementary Fig. 2D). In addition, we also found that *SFRP2* expression was lower in the tumor area in comparison with the NAT area (*p* < 0.001) (Fig. 2E, F). Next, we analyzed the single-cell RNA sequencing of CRC tumors. We found that *SFRP2* expression was mostly expressed in the NAT, although there was a small fraction of the tumor core and tumor border areas that showed a relative increase in *SFRP2* expression. At cell level, we observed that *SFRP2* was predominately expressed in stromal cells (located in the NAT area) and myofibroblasts (located in the tumor core and tumor border areas).

Next, we investigated those variables that can affect the promoter methylation of *SFRP2* in patients with CRC. For that, we conducted a linear regression analysis adjusted for age, sex, and BMI. We found that the location of the tumor (colon vs. rectum) was associated with promoter *SFRP2* methylation (*p* < 0.05) (Table 2). For this purpose, we found that the promoter *SFRP2* methylation in colon was higher than that in rectum (*p* = 0.025) (Supplementary Fig. 1E). In addition, we found that neoadjuvant therapy was strongly associated with *SFRP2* methylation (*p* < 0.001) (Table 2). Accordingly, we found that treated CRC patients had lower promoter *SFRP2* methylation when compared with non-treated CRC patients (*p* < 0.001). We discovered in the Kaplan–Meier plot that low *SFRP2* gene methylation was associated with poorer survival when compared to high methylation status (*p* = 0.027) in non-treated CRC patients (Fig. 2I).

**Table 2:** Association between promoter *SFRP2* methylation and clinicopathological variables under linear and logistic regression

Variables	SFRP2 promoter methylation (continuous)	SFRP2 promoter methylation (Low vs. high)
	β (SD)	OR (95% CI)
Location (Colon vs. rectum)	−0.004 (0.003)*	0.20 (0.06–0.59)**
Lymph node invasion (No vs. yes)	−0.001 (0.001)	0.36 (0.12–0.99)*
Vascular invasion (No vs. Yes)	0.001 (0.002)	1.47 (0.41–5.65)
Metastasis (No vs. yes)	−0.001 (0.001)	0.36 (0.04–1.97)
Neoadjuvant treatment (No vs. yes)	−0.008 (0.009)***	0.05 (0.02–0.20)***
Recurrence (No vs. yes)	−0.001 (0.002)	0.54 (0.18–1.56)
Stage (I + II vs. III + IV)	−0.003 (0.002)	0.31 (0.1–0.86)*

*BMI* Body mass index, *CI* confidence interval, *CRC* colorectal cancer, *HOMAIR* homeostasis model of insulin resistance, *HDL* High-density lipoprotein, *LDL* Low-density lipoprotein, *OR* odds ratio, *SFRP2* secreted frizzled related protein 2, *SD* standard deviation

The multivariate linear and logistic regression analyses were performed using the methylation of the *SFRP2* gene as dependent variable, adjusted by age, sex and BMI. Asterisks indicate significant correlation (*p < 0.05, **p < 0.01, ***p < 0.001)

Furthermore, in order to find other variables that are related to *SFRP2* methylation, we conducted a logistic regression, taking *SFRP2* methylation as at low and high values. We found in the Table 2 that higher promoter *SFRP2* methylation were more likely to be located in the colon (OR = 0.20, 95% CI 0.06–0.59, *p* = 0.005), more likely to be higher in those patients that did not have lymph node invasion (OR = 0.36, 95% CI 0.12–0.99, *p* = 0.05), more likely to be higher in those patients that did not receive neoadjuvant treatment (OR = 0.05, 95% CI 0.02–0.20,* p* < 0.001) and more likely to be higher in those patients in early stage (I + II stages) (OR = 0.31, 95% CI 0.10–0.86, *p* = 0.040).

### SFRP2 decreases cell proliferation and migration in colorectal *cancer* cell

**Fig. 3:**
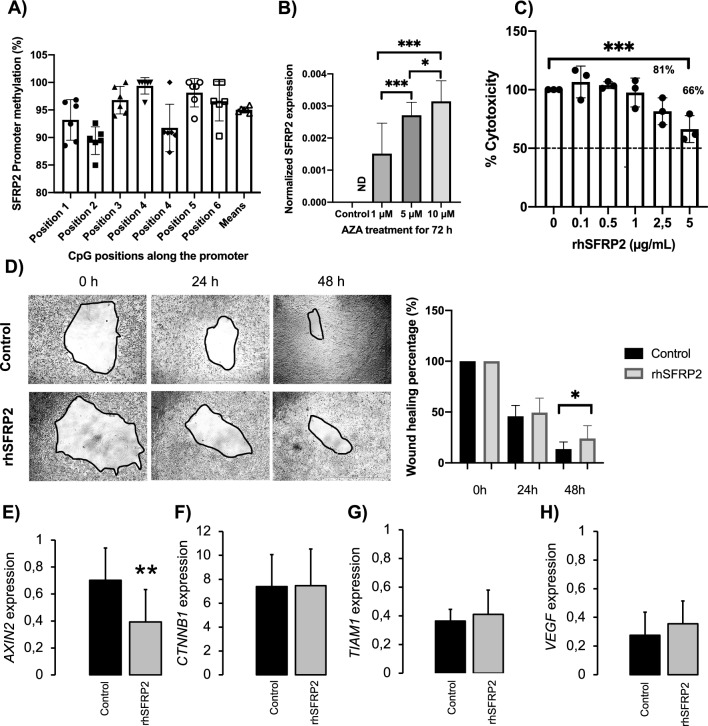
SFRP2 inhibit cell proliferation and migration, through decreasing *AXIN2* expression. **A** promoter methylation of *SFRP2* in HCT116 cells. **B** Cells were seeded in 6-well plates at the concentration of 50.000 cell/well. Cells were treated with 1, 5 and 10 µM of AZA (5-Aza-2-deoxycytidine), during 72 h. *SFRP2* gene expression was measured using qPCR and Probe qPCR kit (Takara, Spain). Gene normalization was used by PPIA. **C** Cells were treated at crescent concentration of rhSFRP2 at 0.1, 0.5, 1, 2.5 and 5 µg/mL of rhSFRP2 during 48 h. **D** wound healing Assay was conducted in the presence of 5 µg/mL of rhSFRP2. **E**–**H** gene expression in cell treated with vehicle or with 5 µg/mL of rhSFRP2 of *AXIN2*, *CTNNB1*, *TIAM1* and *VEGF*. *AZA* azacytidine, *CTNNB1* beta-catenin, *ND* No Detected, *PPIA* Peptidylprolyl Isomerase A, *rhSFRP2* recombinant human secreted frizzled related Protein 2, *TIAM1* T-cell lymphoma invasion and metastasis-inducing protein 1, *VEGR* Vascular endothelial growth factor

We next studied SFRP2 in a cell model, using HCT116 cells. We analyzed the promoter methylation in these cells. We found that the promoter of *SFRP2* gene in HCT116 was fully methylated at 7 CpG sites that we analyzed. The percentages of methylation were 93 ± 3.7% in site 1; 89 ± 2.5% in site 2; 97 ± 2.5% in site 3; 99 ± 1.5% in site 4; 92 ± 4.3% in site 5; 98 ± 2.5% in site 6 and 97 ± 3.6% in site 7, being the mean of all CpG sites 95% (Fig. 3A). Then, we studied whether this fully methylated promoter could affect *SFRP2* expression. For this, we treated HCT116 cells with AZA, an DNMT inhibitor. Before AZA treatment, we did detect *SFRP2* expression in HCT116 cells. However, after AZA treatment, we found that *SFRP2* expression was recovered at a dose-dependent effect, increasing at 1, 5 and 10 µM, respectively (*p* < 0.001) (Fig. 3B).

As *SFRP2* is not expressed in HCT116, we decided to treat the cells with recombinant human SFRP2 (rhSFRP2). After 48 h of treatment, we observed that cell growth was maintained al 100% of cell viability at the concentrations of 0.1, 0.5 and 1 µg/mL of rhSFRP2. At 2.5 µg/mL of rhSFRP2, the cell survival decreased up to 81%, and up to 66% when we treated cells with 5 µg/mL of rhSFRP2, with a significant difference between 5 µg/mL and vehicle (*p* < 0.001). Then, we decided to use 5 µg/mL of rhSFRP2 concentration for the following experiments (Fig. 3C). Next, we evaluated the role of rhSFRP2 in cell migration. At 24 h after scratching cells, we did not observe a significant difference between cells treated with rhSFRP2 and vehicle. However, after 48 h, we observed that cells migrated less when they were treated with 5 µg/mL of rhSFRP2 (*p* = 0.017) (Fig. 3D). Furthermore, we found that treatment with rhSFRP2 decreased the expression of *AXIN2* (*p* < 0.01) (Fig. 3F). No effect was observed in the *CTNNB1*, *TIAM1* and *VEGF* genes (Fig. 3G–I).

## Discussion

In our study, we characterized the expression and DNA methylation levels of *SFRP2* in whole blood, adipose tissue, and colonic tissues, considering different cancer outcomes. We found that *SFRP2* promoter methylation in whole blood is a promising prognostic factor for several cancer outcomes, such as stages, lymph node invasion and CRC recurrence, while *SFRP2* promoter methylation in tumors is a potential predictor of treatment response. Additionally, treatment with rhSFRP2 was able to decrease cell proliferation, likely due to repression of *AXIN2* in the Wnt signaling pathway, indicating its role as a therapeutic tool. The integration of in vitro experiments, clinical data, and bioinformatics analyses provides a comprehensive approach, regarding molecular mechanisms, potential therapeutic targets, and predictive and therapeutic biomarkers. This integration enhances our understanding of the role of SFRP2 in the CRC context. Consequently, SFRP2 is a potential tumor suppressor gene in CRC, which could be recommended as a therapeutic target for cancer clinical practice.

Several studies have evaluated the use of *SFRP2* methylation as a non-invasive biomarker. Using blood as a non-invasive clinical tool, these studies found that *SFRP2* methylation was predominantly hypermethylated in blood samples [27], including circulating tumor cells (CTCs) [28], in serum [29], circulating free DNA [30, 31], and plasma [32, 33]. However, contradictory results were observed, showing low sensitivity and specificity, mainly because most of these studies used the bisulfite PCR method to evaluate the methylation status of *SFRP2*. In our study, utilizing specific *SFRP2* promoter DNA pyrosequencing, we did not observe a significant difference in whole blood between healthy participants and patients. This finding contrasts with that of a meta-analysis [34], underscoring the importance of increasing the sample size to draw definitive conclusions regarding its utility as diagnostic biomarkers. However, we found that low SFRP2 methylation was observed in patients with late-stage disease, lymph node invasion, and recurrence. Similarly, Zhang et al. (2015) found that *SFRP2* methylation in plasma was significantly correlated with TNM stage and lymph node invasion, as observed in our study [33], indicating that *SFRP2* methylation may be a good prognostic biomarker for specific clinical outcomes. In contrast, another study conducted by Bartak et al. (2022) did not find a significant difference in promoter *SFRP2* in cell-free DNA between patients with stable disease and those with recurrence or remission [31]. However, further studies are needed to establish a standardized method to measure *SFRP2* methylation, involving a large number of participants.

On the other hands, multiple studies have reported that *SFRP2* is hypermethylated in tumor tissue from CRC patients [11, 35, 36]. However, the results have shown higher variability. In our study, we determined the global β methylation of the *SFRP2* gene in the tumor and NAT area using DNA array. We found that *SFRP2* methylation in the tumor area was significantly higher than that in the NAT area, but not for *SFRP2* expression, probably due to the sample size. These results were validated using the TCGA-COAD and READ cohorts, and by studying the promoter methylation as reported by our group in a previous study [11]. However, our results did not show that *SFRP2* methylation in the tumor had prognostic value. Controversial results were observed; a study performed by Liu et al. (2019) found that *SFRP2* hypomethylation in the tumor had significantly shorter survival than those with *SFRP2* hypermethylation [36]. In contrast, a study performed by Tang et al. (2011) found that *SFRP2* hypermethylation in the tumor had significantly shorter survival than those negative for *SFRP2* methylation and was an independent prognostic factor [10]. Therefore, further studies are needed to clarify these observations.

We also found that *SFRP2* methylation in tumors is very sensitive to chemotherapy, which could be a potential prognostic factor for therapy monitoring. Treated patients showed lower *SFRP2* methylation than those who were not treated. Indeed, untreated patients with lower *SFRP2* methylation had poor overall survival compared to those with high *SFRP2* methylation. A study conducted by Singh et al. (2018) found that *SFRP2* methylation in peripheral blood plasma decreased in those patients after chemotherapy [37]. In addition, another study found that *SFRP2* was downregulated in liver metastasis CRC patients [38], indicating a sensitive biomarker for treatment response. However, further studies are needed to understand which variables affect *SFRP2* methylation. In our logistic and linear regression analysis, we found that *SFRP2* methylation could be affected by therapy, stage, lymph node invasion, as well as location. Accordingly, we found that SFRP2 methylation was higher in the colon compared to the rectum, as observed by Bagci et al. [39]. Therefore, several clinical outcomes should be taken into account when evaluating the diagnostic and prognostic factors of the SFRP2 gene in CRC. Given the variability in *SFRP2* methylation status observed across studies and tissues, it is crucial to recognize that this variation may be attributed to tissue type and population differences. Therefore, considering multiple confounding biomarkers is imperative to prevent misinterpretation.’ This addition underscores the importance of considering various factors when interpreting *SFRP2* methylation data.

SFRP2 is a member of the SFRP family of proteins involved in the regulation of the Wnt signaling pathway as an antagonist of Wnt proteins. The Wnt pathway is critical in many biological processes, including cancer. Specifically, SFRP2 has a complex role in the development and progression of CRC [40]. Since *SFRP2* is primarily expressed in stromal cells and silenced in tumors, as scRNASeq analysis showed, this phenomenon can elucidate its role as a tumor suppressor gene. In our study, we found that *SFRP2* was silenced in HCT116, and AZA treatment restored *SFRP2* expression in a dose-dependent manner. It is worth to note that DNMT inhibition (through AZA) leads to hypomethylation of CpG sites, resulting in increased accessibility of the gene promoters to transcription factors and other regulatory proteins. This increased accessibility can lead to enhanced gene expression of previously silenced genes. Ectopic rhSFRP2 decreased cell proliferation and migration, probably through decreasing *AXIN2* expression, but not for *CTNNB1*, *TIAM1* and *VEGF*, mainly due to specific action of SFRP2 protein as a Wnt antagonist. AXIN2 act as negative regulators of the Wnt signaling pathway by promoting the phosphorylation and degradation of β-catenin. Several studies have demonstrated similar findings in different cancer contexts, such as in MG63 cells (osteosarcoma) [41], SW480 cells (colorectal cell line) [42], At-T20 cells (mouse pituitary tumor cells) [43], as well as CCLP-1 and QBC939 cells (Cholangiocarcinoma).

This effect was mainly observed through the increase in the expression of phosphorylated-β-catenin and phosphorylated-GSK3β in stem cells of apical papilla [44], and melanocytes [45], as well as decreased *AXIN2* expression [44], as observed in our study. Another study found that treatment with rhSFRP2 in MG63 cells increased RhoA activity, which decreased the effects induced by Wnt5A [41]. Indeed, rhSFRP2 decreased RhoA activity induced by Wnt5A in both U251 and T98MG cells (glioblastoma) [46]. Furthermore, a study conducted by Kim et al. (2018) showed that SFRP2 can act through the canonical Wnt/β-catenin pathway in an independent manner in iPSC-derived MSCs [47], suggesting new roles in the Wnt pathway. Overall, the effects of *SFRP2* overexpression in HCT116 cells suggest that this protein may have a tumor-suppressive function in colorectal cancer. rhSFRP2 could act as therapeutic target, through its ability to attenuate the Wnt pathway. Given the predominant silencing of *SFRP2* observed in colorectal tumors, indicative of its role as a tumor suppressor gene, the rhSFRP2 model provides valuable insights into its impact on pivotal pathways. Specifically, our findings shed light on SFRP2's modulation of the Wnt pathway through its interaction with the *AXIN2* gene, presenting novel therapeutic possibilities. However, the role of SFRP2 in cancer cells is multifaceted and likely contingent upon the specific cellular environment and context. Our study highlights several biomedical applications of SFRP2 in the context of CRC. *SFRP2* methylation patterns can be used in early detection, disease progression assessment, and therapy monitoring. Further insights into disease mechanisms can be gained by deepening the characterization of SFRP2 in mice and other in vitro models, thereby uncovering the therapeutic potential of rhSFRP2. Further studies are needed to fully elucidate the mechanisms underlying these effects and to determine the potential clinical implications of SFRP2 as a therapeutic target in cancer therapy [48, 49].

There are several limitations that worth to mention related this study. Firstly, this research is based on an observational and prospective study with limited sample size, that need to be validated in future research. Also, longitudinal studies are needed, particularly in standardizing methods for measuring *SFRP2* methylation and understanding its variable roles in cancer. Further molecular and mechanistic research on SFRP2 is needed to provide a comprehensive understanding on its role in colorectal carcinogenesis. Additionally, the use of specific *SFRP2* promoter DNA pyrosequencing and Infinium Human Methylation 450 K bead chip technology offers several advantages, including high sensitivity and specificity in detecting DNA methylation patterns, when compared to other methods such as Methyl-Specific PCR. However, techniques like bisulfite-DNA sequencing provide extensive coverage, capturing CpG sites not included in this study. It is important to acknowledge potential limitations such as the requirement for specialized equipment and expertise, as well as the necessity for rigorous result validation.

## Conclusion

Studies have shown that SFRP2 plays a complex role in the development and progression of CRC. In our study, we found that *SFRP2* methylation in whole blood is a potential biomarker for several cancer outcomes, while *SFRP2* methylation in tumor tissue is a promising predictor for therapy response, suggesting its potential use in clinical practice. In a CRC cell model, *SFRP2* was mainly found to be suppressed, and its expression was restored by DNA demethylation, indicating epigenetic silencing. Treatment with rhSFRP2 was able to decrease cell proliferation and migration, suggesting a role as a tumor suppressor gene, although previous steps in other biological models are needed. However, the exact mechanisms by which SFRP2 influences CRC development and progression are still not fully understood. Nonetheless, our findings suggest that SFRP2 may be a promising target for the development of novel therapeutic approaches for CRC.

### Supplementary Information

Supplementary material 1: Supplementary Figure 1. We measured the normalized A) *SFRP2* expression in blood sample from healthy participants (N=28) and patients with CRC (N=27). B) Kaplan-Meier curve comparing the median of the *SFRP2* promoter methylation as low and high methylation. C) We measured the promoter methylation of *SFRP2* gene from healthy participants and patients with CRC in early stage (I+II) and late stage (III+IV). D) SFRP2 expression in adipose tissue healthy participants (N=54) and patients with CRC (N=64) and F) *SFRP2* methylation in adipose tissue healthy participants (N=56) and patients with CRC (N=41), Kaplan-Meier curve comparing the median of the E) *SFRP2* expression in whole blood as low and high expression, *SFRP2* expression in adipose tissue as low and high expression and G) promoter *SFRP2* methylation in adipose tissue as low and high expression. The significance of differences is evaluated with the Log-rank test. Asterisks indicate significant differences between the groups according to the Mann Whitney test (*p<0.05, **p<0.01, ***p<0.001).

Supplementary material 2 A) Kaplan-Meier curve comparing the median of the SFRP2 global β methylation as low and high methylation in A) our population, C) TCGA-COAD and D) TCGA-READ, as well as by studying B) promoter methylation. The significance of differences is evaluated with the Log-rank test. E) Promoter SFRP2 methylation in in tumor by comparing colon (N=30) vs rectum (N=51). Asterisks indicate significant differences between the groups according to the Mann Whitney test (*p<0.05, **p<0.01, ***p<0.001).

Supplementary material 3: Table 1. Colorectal cancer outcome prediction using *SFRP2* methylation as predictive biomarker performed by ROC curve analysis

## Data Availability

The datasets generated and/or analyzed during the current study are not publicly available due the participants were anonymized but are available from the corresponding author on reasonable request.
