# Supplementary figures and images for: Identification of epigenetic silencing of the SFRP2 gene in colorectal cancer as a clinical biomarker and molecular significance
Source: J Transl Med. 2024 May 27;22:509. doi: 10.1186/s12967-024-05329-x (PMC11129357; doi:10.1186/s12967-024-05329-x)

# Whole blood

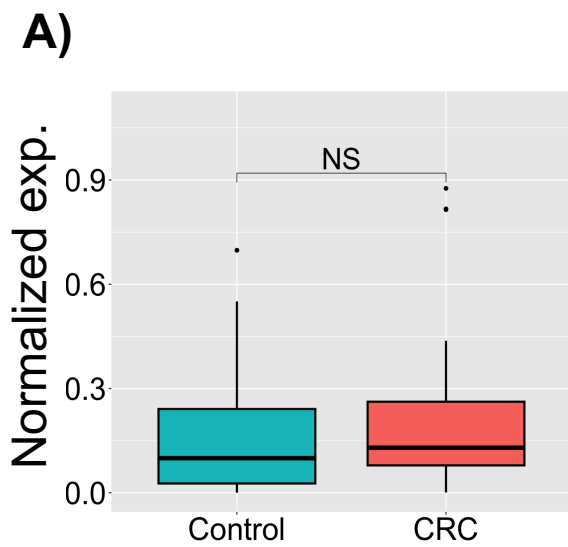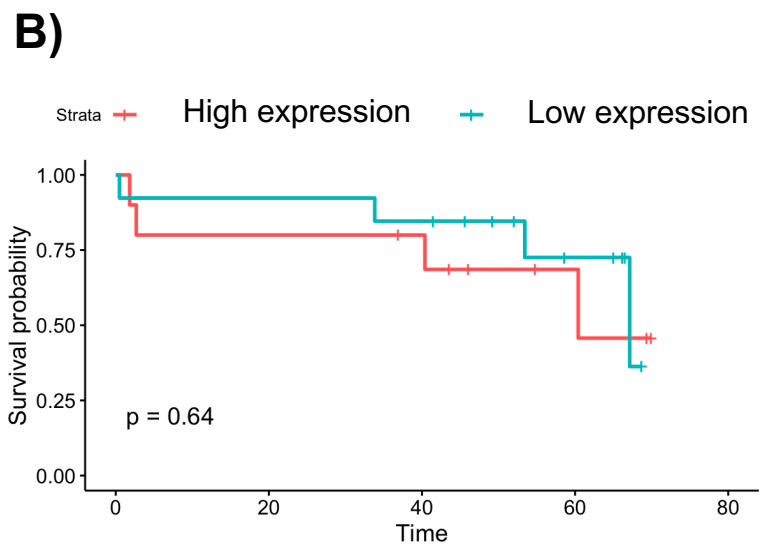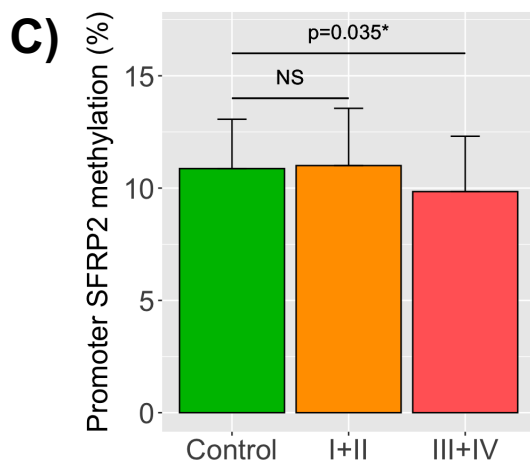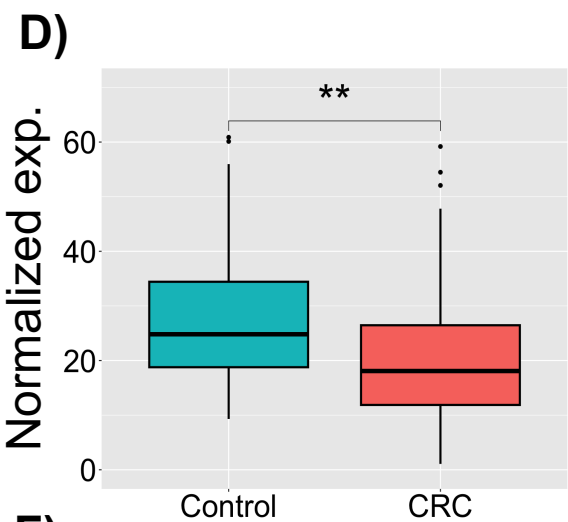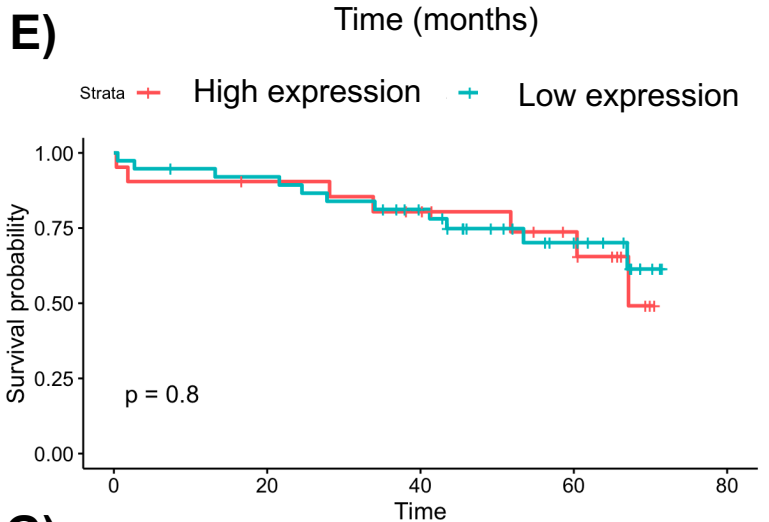

# Adipose tissue

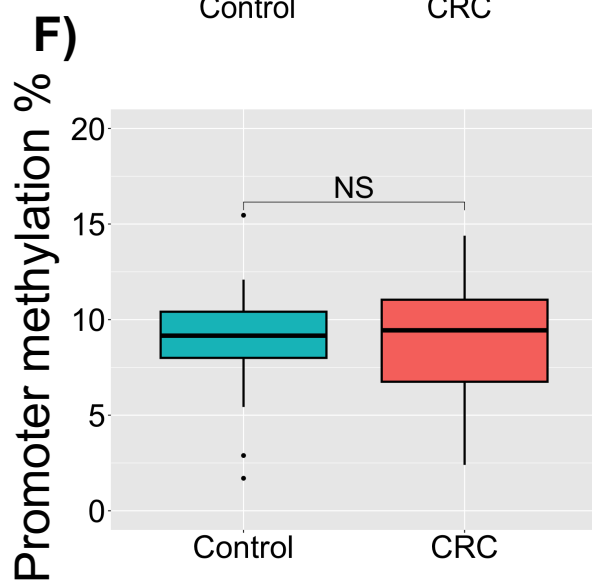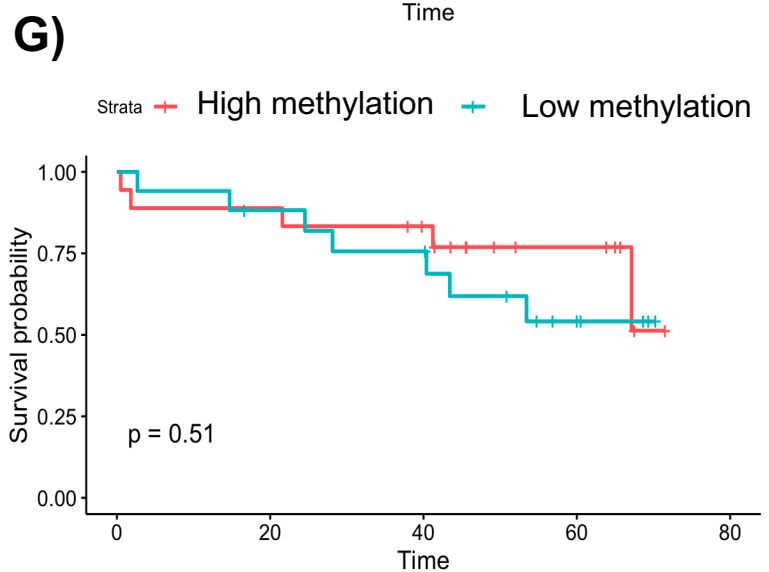

Supplement: Supplementary file 1 — Supplementary material 1: Supplementary Figure 1. We measured the normalized A) SFRP2 expression in blood sample from healthy participants (N=28) and patients with CRC (N=27). B) Kaplan-Meier curve comparing the median of the SFRP2 promoter methylation as low and high methylation. C) We measured the promoter methylation of SFRP2 gene from healthy participants and patients with CRC in early stage (I+II) and late stage (III+IV). D) SFRP2 expression in adipose tissue healthy participants (N=54) and patients with CRC (N=64) and F) SFRP2 methylation in adipose tissue healthy participants (N=56) and patients with CRC (N=41), Kaplan-Meier curve comparing the median of the E) SFRP2 expression in whole blood as low and high expression, SFRP2 expression in adipose tissue as low and high expression and G) promoter SFRP2 methylation in adipose tissue as low and high expression. The significance of differences is evaluated with the Log-rank test. Asterisks indicate significant differences between the groups according to the Mann Whitney test (*p<0.05, **p<0.01, ***p<0.001). [file 12967_2024_5329_MOESM1_ESM.pdf]
