# Supplementary material for: Identification of epigenetic silencing of the SFRP2 gene in colorectal cancer as a clinical biomarker and molecular significance
Source: J Transl Med. 2024 May 27;22:509. doi: 10.1186/s12967-024-05329-x (PMC11129357; doi:10.1186/s12967-024-05329-x)
Supplement: Supplementary file 2 — Supplementary material 2 A) Kaplan-Meier curve comparing the median of the SFRP2 global β methylation as low and high methylation in A) our population, C) TCGA-COAD and D) TCGA-READ, as well as by studying B) promoter methylation. The significance of differences is evaluated with the Log-rank test. E) Promoter SFRP2 methylation in in tumor by comparing colon (N=30) vs rectum (N=51). Asterisks indicate significant differences between the groups according to the Mann Whitney test (*p<0.05, **p<0.01, ***p<0.001). [file 12967_2024_5329_MOESM2_ESM.pdf]

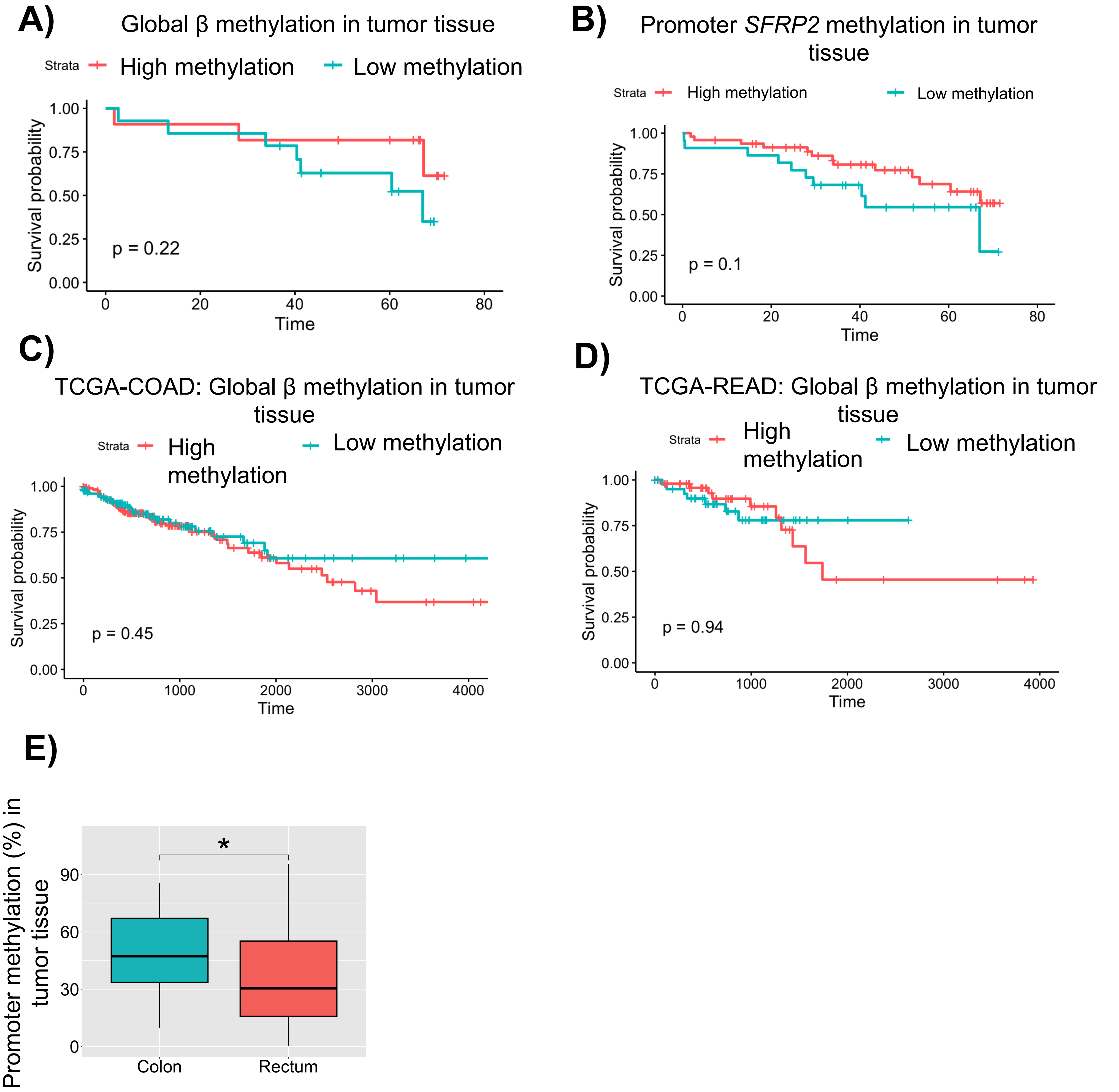
A)
Global β methylation in tumor tissue
Strata
High methylation
Low methylation
Survival probability
p = 0.22
Time
B)
Promoter SFRP2 methylation in tumor tissue
Strata
High methylation
Low methylation
Survival probability
p = 0.1
Time
C)
TCGA-COAD: Global β methylation in tumor tissue
Strata
High methylation
Low methylation
Survival probability
p = 0.45
Time
D)
TCGA-READ: Global β methylation in tumor tissue
Strata
High methylation
Low methylation
Survival probability
p = 0.94
Time
E)
Promoter methylation (%) in tumor tissue
*
Colon
Rectum
