# Supplementary material for: Identification of epigenetic silencing of the SFRP2 gene in colorectal cancer as a clinical biomarker and molecular significance
Source: J Transl Med. 2024 May 27;22:509. doi: 10.1186/s12967-024-05329-x (PMC11129357; doi:10.1186/s12967-024-05329-x)
Supplement: Supplementary file 3 — Supplementary material 3: Table 1. Colorectal cancer outcome prediction using SFRP2 methylation as predictive biomarker performed by ROC curve analysis [file 12967_2024_5329_MOESM3_ESM.docx]

**Supplementary Table 1.** Colorectal cancer outcome prediction using *SFRP2* methylation as predictive biomarker performed by ROC curve analysis.

| **Model** | **AUC** | **Sensitivity** | **Specificity** | **Accuracy** | **Threshold** |
| --- | --- | --- | --- | --- | --- |
|  |  |  |  |  |  |
| Model 1 |  |  |  |  |  |
| Stage | 0.653 (0.510 – 0.796) | 0.467 | 0.827 | 0.736 | 0.579 |
| Lymph node invasion | 0.689 (0.545 – 0.832) | 0.692 | 0.687 | 0.642 | 0.470 |
| Recurrence | 0.699 (0.551 – 0.848) | 0.526 | 0.857 | 0.625 | 0.393 |
| Model 2 |  |  |  |  |  |
| Stage | 0.752 (0.639 – 0.866) | 0.500 | 0.941 | 0.900 | 0.642 |
| Lymph node invasion | 0.738 (0.618 – 0.856) | 0.576 | 0.868 | 0.791 | 0.559 |
| Recurrence | 0.764 (0.654 – 0.874) | 0.956 | 0.500 | 0.468 | 0.227 |
| Model 3 |  |  |  |  |  |
| Stage | 0.879 (0.782 – 0.975) | 0.704 | 0.920 | 0.905 | 0.664 |
| Lymph node invasion | 0.844 (0.736 – 0.951) | 0.833 | 0.786 | 0.769 | 0.473 |
| Recurrence | 0.821 (0.700 – 0.942) | 0.722 | 0.833 | 0.684 | 0.429 |
|  |  |  |  |  |  |

Receiver operating characteristic curves of the model including the clinical and *SFRP2* methylation variables.

- Model 1: This model includes SFRP2 methylation in whole blood.

- Model 2: This model includes age, sex, body mass index, HDL, triglycerides and cancer location.

- Model 3: This model includes Model 1 + Model 2.

**Abbreviations**. HDL: high density lipoprotein; ROC: receiver operating curve; SFRP2: Secreted Frizzled-related protein 2.
